# The impact of the COVID-19 pandemic era on children with primary headache: a questionnaire survey study and literature review

**DOI:** 10.3389/fped.2023.1179979

**Published:** 2023-07-10

**Authors:** So Yeon Yoon, Hye Min Kim, Yoon Young Yi

**Affiliations:** ^1^ Department of Pediatrics, Hallym University and Kangdong Sacred Heart Hospital, Seoul, Republic of Korea; ^2^ Department of Pediatrics, College of Medicine, Soonchunhyang University Bucheon Hospital, Bucheon, Republic of Korea

**Keywords:** COVID-19, primary headache, children, migraine, pandemic

## Abstract

**Background:**

The coronavirus disease (COVID-19) pandemic has resulted in individual isolation and secondary problems, especially in children. Research on the effect of the social isolation on children with primary headache is limited. This study aimed at exploring the effects of environmental changes caused by COVID-19 on headache in children.

**Methods:**

This cross-sectional survey study enrolled school-aged children (age, 8–16 years) with headache who were able to complete the questionnaire from a Pediatric Headache Clinic between January 2021 and December 2022. Headache diaries for all patients were in their medical records and two questionnaire responses were requested at a 3-month interval. The questionnaires included headache type, frequency, previous medical conditions, family history, Pediatric Migraine Disability Assessment scores (PedMIDAS) scores, changes in daily life after COVID-19, and factors that aggravated headaches associated with social distancing.

**Results:**

We identified 35 patients who were diagnosed with primary headache and continued to visit our outpatient clinic for at least 3 months. Among them, 33 (15 males and 18 females) patients responded to the first survey. The average age (±SD) of patients was 12.5 ± 1.9 years. PedMIDAS scores were not affected by the COVID-19 infection history. Prolonged use of masks and increased use of digital devices were reported as the most common factors that aggravated headache during the pandemic era.

**Conclusion:**

COVID-19 did not affect in worsening primary headache in children. However, the pandemic can introduce various changes in daily life, which in turn can affect the management of headache. By gathering feedback regarding the thoughts of the patients on the impact of the current pandemic environment, patient counseling on the precautions and management can be conducted in advance in the case of repeated lockdown in the future.

## Introduction

The COVID-19 outbreak, started in Wuhan, China in December 2019 and spread rapidly worldwide. It was declared as a pandemic by World Health Organization in March 2020 (1). Since it is transmitted through respiratory droplets, isolation of the affected person and the person in contact is important to prevent further transmission. The quarantine strategy has been activated to prevent virus spread all over the world. In South Korea, social distancing has also been implemented under the control of the Korea Disease Control and Prevention Agency. Students who lived in groups at school were mostly affected by these daily changes. After the first COVID-19 outbreak in February 2020, elementary, middle and high school students were completely banned from attending school. From mid-April 2020, online classes began sequentially, starting with high school students. Between November 2020 and April 2022, most schools were closed, according to the ratio of the number of confirmed cases to the number of people in self-quarantine. Until the social distancing ended, students relied on irregular school days and online classes (Supplementary Data 1). During this long period, students had to attend school every other week or every other day in a limited number of students per class, while following many rules related to their daily activities, such as sitting at a distance from each other, not talking to each other during breaks, going to the restroom in order, and not eating at school. Moreover, if a child or a family member was affected by COVID-19, a long quarantine duration was mandated. Although social distancing has ended, wearing a mask was maintained indoors when living with others until January 2023.

The social isolation has had negative effects on children and adolescents with various neurological diseases, such as epilepsy (2, 3), neurodevelopmental disorders (NDD) (4–6), and psychological problems (7, 8). Dal-Pai et al. performed systematic review of the impact of the pandemic on children and adolescents with epilepsy. It showed that they had difficulty learning remotely, had worse sleep and behavior, and spent more time using electronic devices during the pandemic. Kawaoka et al. showed that children with autism spectrum disorder (ASD), attention-deficit hyperactivity disorder (ADHD), and intellectual disorder had increases in behavioral problems after the onset of school closure by using Child Behavior Checklists about before and after school closure (6). It was found that externalizing and aggressive behavior increased in all children with NDDs (6, 8). Zhou et al. (7) and Duan et al. (8) reported high prevalence of anxiety and depression among children and adolescents during the pandemic.

Primary headache is a frequent neurological symptom in school-aged children. The reported prevalence of migraine and tension-type headache (TTH) is 11% and 17%, respectively. In addition, 62% of primary headaches occur in children and adolescents (9). The frequency and duration of headache have a significant impact on the quality of life of children (10). Conversely, environmental and social influences may contribute to the aggravation of primary headache. Patients with primary headache disorders perceive external factors such as, stress, sleep, and changes in their everyday routine, as common triggers of their headache attacks (11). Frequent school closure and dramatic lifestyle change due to COVID-19 may affect the course of primary headaches in children. The COVID-19 pandemic can also result in individual isolation and secondary problems due to increased smartphone use and irregular life styles and act as a worsening factor for headaches, especially in children. The social isolation situation is a seriously abnormal living environment for children, which has never happened in the past, so studies on the effect of a changed lifestyle during this limited period on primary headaches are absolutely necessary. The aim of this study was to analyze patients' thoughts through a survey on the impact of environmental changes caused by COVID-19 on primary headaches in children.

## Materials and methods

### Study design and questionnaires

**Figure 1:**
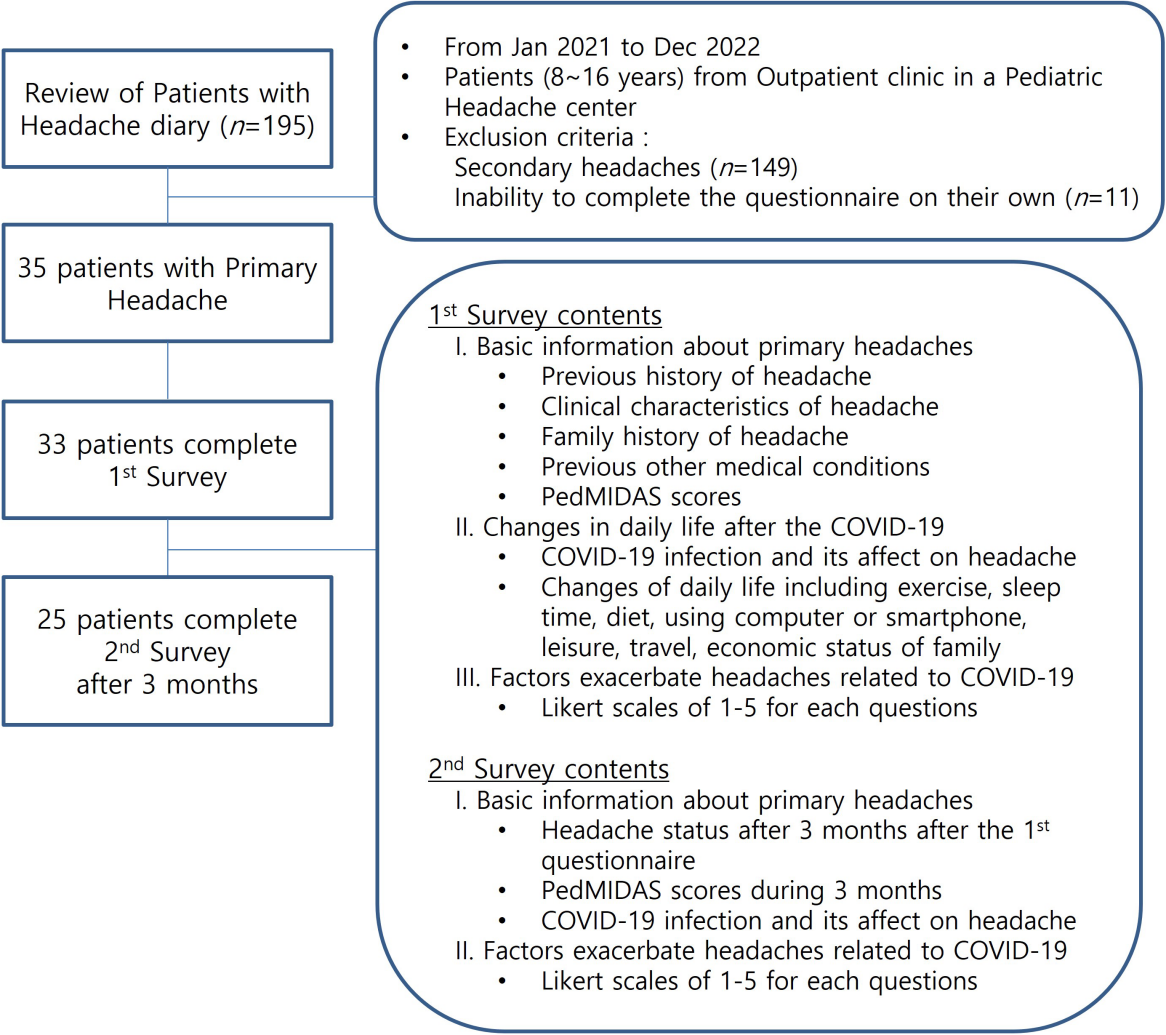
Study flow diagram: review the patients with headache and cross-sectional surveys.

This study was conducted at the Pediatric Headache Clinic of Kangdong Sacred Heart Hospital between January 2021 and December 2022. A total of 195 patients from the outpatient clinic were clinically reviewed for headache. Eligible participants were patients: (1) aged 8–16 years and (2) having primary headache according to the International Classification of Headache Disorders-3 (ICHD-3). The exclusion criteria were: (1) the presence of secondary headache and (2) inability to complete the questionnaire (Figure 1). Third-grade or older elementary school students, who had an understanding of the survey items, were asked to fill out the questionnaire appropriately in the absence of underlying diseases or intellectual disabilities. Written informed consent was obtained from all the patients who completed the questionnaire, and the study was approved by the Institutional Review Board of Kangdong Sacred Heart Hospital (IRB no.2021-09-006). A retrospective medical chart review was performed to confirm the precise diagnosis of primary headache, the presence of previous diagnostic tests such as magnetic resonance imaging (MRI) and electroencephalogram (EEG), and the combined psychological problems with Children's Depression Inventory (CDI) and Revised Manifest Anxiety Scale for Children (RCMAS).

The first questionnaire consisted of 3 categories and 36 items. The five items of the first category were, patient information (Q1–3), the headache history of the patient (Q4–9), clinical characteristics of the current headache (e.g., headache intensity, duration, and the presence of photophobia or phonophobia, medications for headache) (Q10–18, Q25–27), previous medical history (Q28–29), family headache history (Q30), and the Pediatric Migraine Disability Assessment (Ped-MIDAS) (Q19–24) (12). In the second category, questions were embodied about whether or not they were infected with COVID-19 and the impact of the COVID-19 pandemic situation on their daily lives (Q31–34). Finally, in the third category, the patients were asked to provide their opinion on the factors aggravating primary headache in the COVID-19 pandemic situation by choosing a score in the range of 1–5 on a Likert scale (Q35–36). The second questionnaire consisted of 2 categories and 16 items, where the first and third categories of the first questionnaire were repeated for the same patients after 3 months. We have attached the original Korean questionnaires to the supplementary material (Supplementary Data 2, 3).

### Statistical analysis

The distribution of continuous variables is described using the mean ± standard deviation (SD). Categorical variables are presented as percentages (%) and frequencies (*n*). Differences between groups were analyzed as nominal variables using the *χ*^2^ test or Fisher's exact test, as appropriate. Paired Student's *t*-test was used to compare the variables before and after the pandemic. Data were assessed using Likert scales and analyzed on an equal continuous scale. Statistical analyses were performed using SPSS Statistics for Windows version 21.0 (IBM Corporation, Armonk, NY, USA). A *p*-value <0.05 was considered statistically significant.

## Results

### Demographics and clinical characteristics

A total of 33 children with primary headache were enrolled in the 1st questionnaire. Three months later, 25 patients were included in the 2nd survey (Figure 1). Table 1 shows the demographics and clinical characteristics of the patients. Their mean age was 12.5 ± 1.9 years and the male to female ratio was 1:1.2. Equal numbers of elementary and middle school students were enrolled (*n *= 16 each) and only one high school student was enrolled. The average age at which the first headache occurred was 10.5 ± 2.6 years. Thirty-six percent of the participants had their first headache before January 2020. Twenty-three patients were diagnosed with migraine, while 10 had TTH. The average headache pain score was 6.2 ± 1.2 points. The duration of headache was <2 and ≥2 h in 69.7% and 30.3% of the patients, respectively. The frequency of headache was widely distributed, and six patients experienced daily headache. Nausea and/or vomiting (54.5%) was the most common associated symptom, and 11 patients reported auras. Acute and preventive treatments were administered to 78.8% and 51.5% of the patients, respectively. Family history of headache was present in 30% of the patients. Brain MRI and EEG were performed in 22 and 5 patients, respectively; and there were no specific findings. Until the time of conducting the first survey, 19 patients were infected with COVID-19 and 21 patients experienced self-isolation through contact with an infected person. Twenty-three patients had medical records of CDI and RCMAS. The average score of CDI was 17.5 ± 10.6, and RCMAS was 12.2 ± 9.7. Seven patients (30.4%) exceeded the CDI clinically significant cutoff point for increased self-reported depression (CDI score >15), and 15 patients (65.2%) exceeded RCMAS for self-reported anxiety (RCMAS score >15) (13).

**Table 1:** Demographics and clinical characteristics of the participants (*n *= 33).

Clinical characteristics	
Age mean (SD), year	12.52 (1.86)
Male:Female	15: 18
Age at headache onset, mean (SD), year	10.38 (2.62)
First headache before January 2020, *n* (%)	12 (36)
Current education status, *n* (%)	
Elementary school	16 (48.5)
Middle school	16 (48.5)
High school	1 (3)
Brain MRI, *n* (%)	22 (66.7)
EEG, *n* (%)	5 (15.2)
Family history of headache, *n* (%)	10 (30.3)
History of COVID-19 infection, *n* (%)	19 (57.6)
Isolation through contact with a person infected with COVID-19, *n* (%)	21 (63.6)
ICHD-3 classification of primary headache, *n* (%)	
Migraine	23 (69.7)
M:F	1:2.3
TTH	10 (30.3)
M:F	4:1
Pain severity (scale, 0–10 scales), mean (SD)	6.24 (1.2)
Headache characteristics, *n* (%)	
Pulsating	19 (57.6)
Pressing	10 (30.3)
Stabbing	3 (9)
Others	1 (3)
Headache medication, *n* (%)	
Acute	26 (78.8)
M:F	11:15
Preventive	17 (51.5)
M:F	4:13
Headache frequency, *n* (%)	
<1 per month	6 (18.2)
1–3 per month	7 (21.2)
1–2 per week	5 (15.2)
2–3 per week	8 (24.2)
>3 per week	1 (3)
Daily	6 (18.2)
Headache duration, *n* (%)	
<few minutes	4 (12.1)
Few minutes–2 h	19 (57.6)
2–12 h	8 (24.2)
12–24 h	2 (6.1)
24–72 h	0
>72 h	0
Auras, *n* (%)	
Visual	8 (24.2)
Sensory	2 (6)
Speech disturbance	1 (3)
Associated symptoms, *n* (%)	
Nausea/Vomiting	18 (54.5)
Photophobia	11 (33.3)
Phonophobia	16 (48.5)
Osmophobia	10 (30.3)
CDI (*n *= 23)	17.5 ± 10.6
RCMAS (*n *= 23)	12.2 ± 9.7

SD, standard deviation; MRI, magnetic resonance imaging; EEG, electroencephalogram; COVID-19, coronavirus disease; ICHD, international classification of headache disorders; TTH, tension type headache; CDI, children's depression inventory; RCMAS, revised children's manifest anxiety scale.

### Ped-MIDAS and trigger factors

The Ped-MIDAS constitutes answers to the number of days of absence, early leaves, and functional decline over the preceding 3 months and can be used as an indicator of the impact of headache. In the first questionnaire, the average of the total Ped-MIDAS score of the 33 patients was 32.64 ± 51.65 points. When the total Ped-MIDAS score of each patient was classified to a grade in range of 1–4, most students were in grades 1 and 2 (Table 2). Higher scores were observed in female students and middle and high school rather than elementary school students, and the patients more often experienced migraine than TTH. However, there was no statistically significant difference between the past history of COVID-19 and Ped-MIDAS scores. Stress induced by schoolwork was the highest (54.5%) trigger of primary headache. Lack of sleep or sleep irregularity, interpersonal stress, and loud noise were experienced by 57.6%, 30.3%, and 33.3% of the patients with primary headache, respectively (Table 3). The changes in the headache patterns after 3 months, as reported in the 2nd questionnaire, are shown in Table 4. The total Ped-MIDAS scores and pain severity were reduced by 76% and 80%, respectively. And school absence rate also fell from 63.6% to 16%. However, the trend toward a decreased in headache frequency was not clear in our data. During the 3-month period, one patient was infected with COVID-19, and two patients were quarantined after being in contact with infected people.

**Table 2:** Correlation between Ped-MIDAS and headache characteristics (*n *= 33).

Variables	Ped-MIDAS	*p*-value
Total Summary score, (mean ± SD)	32.64 ± 51.65	
Grade 1 (0–10), *n* (%)	13 (39.4)	
Grade 2 (11–30), *n* (%)	12 (36.4)	
Grade 3 (31–50), *n* (%)	3 (9)	
Grade 4 (≥51), *n* (%)	5 (15.2)	
School absence, *n* (%)	21 (63.6)	
Sex, (mean ± SD)		
Male	11.27 ± 9.6	
Female	50.44 ± 64.8	0.021*
Headache type, (mean ± SD)		
Migraine	43.65 ± 58.7	
Tension-type headache	7.30 ± 5.9	0.007*
History of COVID-19 infection, (mean ± SD)		
Yes	25.16 ± 43.5	
No	42.79 ± 61.3	0.341
Current status of Education, (mean ± SD)		
Elementary school	22.5 ± 42.2	
Middle/High school	44.81 ± 59.7	0.232
Pain Severity (scale, 0–10), (mean ± SD)		
Mild (1–3)	0	
Moderate (4–6)	36.52 ± 55.5	
Severe (7–10)	20.5 ± 51.7	0.454
Headache frequency, (mean ± SD)		
<1 per month	9.17 ± 7.9	
1–3 per month	17.43 ± 16.8	
1–2 per week	51.6 ± 97.8	
2–3 per week	28.38 ± 36.1	
>3 per week	17.0	
Daily	66.33 ± 66.4	0.408
Headache duration, (mean ± SD)		
< few minutes	78 ± 100.6	
Few minutes–2 h	16.63 ± 21.8	
2–12 h	52 ± 65.3	
12–24 h	16.5 ± 2.1	
24–72 h	0	
>72 h	0	0.094

SD, standard deviation.

* *p *< 0.05 was considered statistically significant.

**Table 3:** Triggers of primary headache.

Triggers	Total (*n *= 33), *n* (%)	Migraine (*n *= 23), *n* (%)	TTH (*n *= 10), *n* (%)
Academic stress	18 (54.5)	11 (47.8)	7 (70)
Interpersonal stress	10 (30.3)	8 (34.8)	2 (20)
Sleep problems	19 (57.6)	15 (65.2)	4 (40)
Eating problems	3 (9.1)	1 (4.3)	2 (20)
Irregular meals	2 (6.1)	0	2 (20)
Strong odors	5 (15.2)	3 (13)	2 (20)
Noise	11 (33.3)	10 (43.5)	1 (10)
Weather changes	3 (9.1)	3 (13)	0
Sun exposure	2 (6.1)	2 (8.7)	0
Exercise	4 (12.1)	3 (13)	1 (10)
Abnormal humidity	2 (6.1)	1 (4.3)	1 (10)
Specific food intake	1 (3)	1 (4.3)	0
Coffee	1 (3)	1 (4.3)	0
Alcohol	0	0	0
Smoking	0	0	0
Menstruation	0	0	0

TTH, tension type headache.

**Table 4:** Changes in the headache patterns after 3 months in the 2nd survey (*n *= 25).

Variables	
Age (mean ± SD), years	13.04 ± 2.0
Male:Female	12:13
ICHD-3 classification of primary headache, *n* (%)	
Migraine	18 (72)
TTH	7 (28)
Headache medication, *n* (%)	
Acute	15 (60)
Preventive	13 (52)
Pain severity (scale, 0–10), *n* (%)	
Increased	3 (12)
Unchanged	2 (8)
Decreased	20 (80)
Ped-MIDAS score	
Total Summary score	8.24 ± 10.7
Increased, *n* (%)	6 (24)
Unchanged, *n* (%)	0
Decreased, *n* (%)	19 (76)
School absence, *n* (%)	4 (16)
Headache frequency, *n* (%)	
Increased	9 (36)
Unchanged	5 (2)
Decreased	11 (44)
COVID-19 infection during 3 months, *n* (%)	1
Isolation through contact with a person infected with COVID-19 during 3 months, *n* (%)	2

SD, standard deviation; ICHD, international classification of headache disorders; TTH, tension type headache; Ped-MIDAS, pediatric migraine disability assessment.

### COVID-19 pandemic and daily lifestyle changes experienced by children

The impact of COVID-19 pandemic on the changing daily lives of students is presented in Table 5. The average mask wearing time was 46.7 ± 18.2 h/week. During the pandemic, the school class hours decreased from an average of 5.9 ± 1.2 to 5 ± 1.9 h/day. The class time of the private tutoring centers, where the Korean students usually go to after school, also decreased from an average of 8.6 ± 10.8 to 5.5 ± 9.4 h/week. The smartphone usage time increased from an average of 2.9 ± 2.1 to 4.2 ± 3.1 h/day. Moreover, the frequency of going out decreased from an average of 4.1 ± 2.1 to 2.4 ± 2.1 times/week, and leisure activities, such as traveling and watching performances, decreased from an average of 2.8 ± 2.5 to 1.3 ± 1.3 times/month. In addition, the exercise time decreased by 55%, while the weight of the students increased by 33%. Thirty-six percent of the students complained about facing difficulties in online classes. However, financial problems were reported in only 6% of the cases.

**Table 5:** Impact of the COVID-19 pandemic on daily life activities in children.

Variables	*Before* Pandemic (mean ± SD)	*After* Pandemic (mean ± SD)	*p*-value	Variables, *n* (%)
Wearing a mask	No use	Total 46.7 ± 18.2 h/week 6.1 ± 1.0 days/week 7.5 ± 2.2 h/day		Exercise time
Class time				Increased 2 (6) Unchanged 13 (39) Decreased 18 (55)
At school	5.9 ± 1.2 h/day	5.0 ± 1.9 h/day^a^	0.010*	Weight
At private tutoring centers	8.6 ± 10.8 h/week	5.5 ± 9.4 h/week^a^	0.078	Increased 11 (33) Unchanged 20 (61) Decreased 2 (6)
Smartphone usage time	2.9 ± 2.1 h/day	4.2 ± 3.1 h/day	0.000*	Difficulty in online classes
Other digital devices usage time	1.7 ± 1.7 h/day	2.1 ± 2.3 h/day	0.125	Yes 12 (36) No 21 (64)
Frequency of going out	4.1 ± 2.1 times/week	2.4 ± 2.1 times/week	0.002*	Financial problem
Frequency of leisure time	2.8 ± 2.5 times/month	1.3 ± 1.3 times/month	0.000*	Yes 2 (6) No 31 (94)

COVID-19, coronavirus disease; SD, standard deviation.

^a^ Online classes.

* *p *< 0.05 was considered statistically significant.

**Figure 2:**
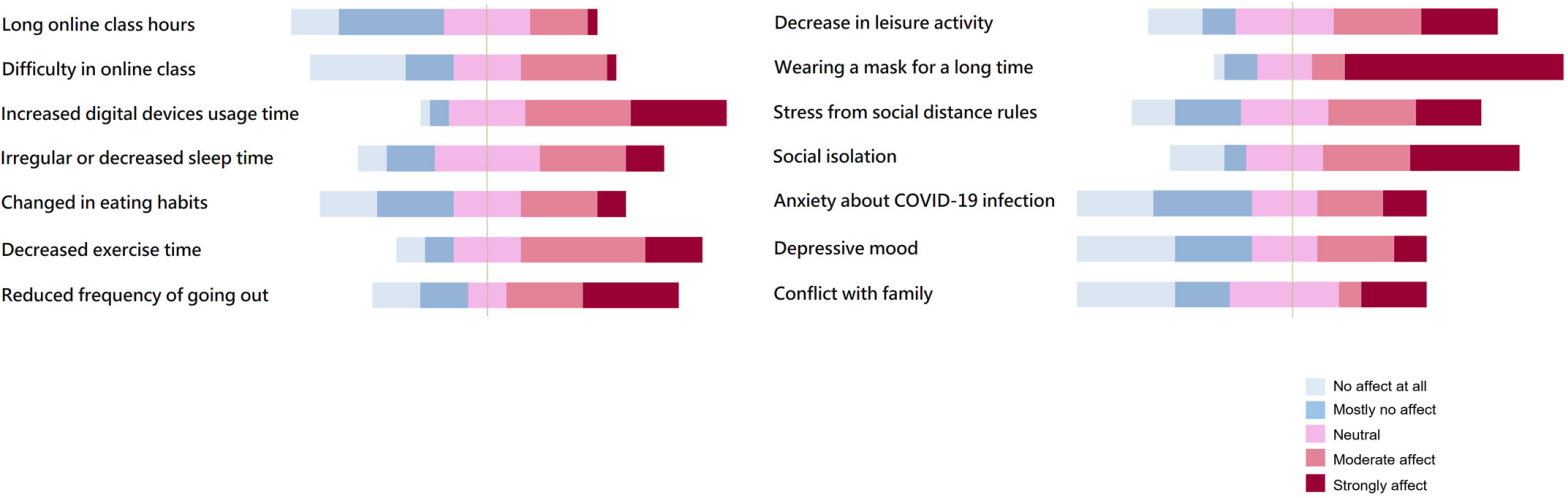
Risk factors of worsening primary headache on the COVID-19 pandemic environment.

Patients were asked to rate the degree to which they contributed to their headache exacerbation on a scale of 1–5 (Figure 2). The most selected factor among all the items was the long-term use of masks, “strongly affect (62.5%)”. The sum of the response rates of “moderate affect” and “strongly affect” were showed in the following orders; the increased digital devices usage time (65.6%), decreased exercise time (59.4%), reduced frequency of going out (56.3%), social isolation (56.3%), decreased leisure activity (46.9%), stress from social distance rules (43.8%), and irregular or decreased sleep time (40.6%) (Table 6).

**Table 6:** The distribution of the answers for the risk factors of worsening primary headache by the COVID-19 pandemic environment (*n* = 32).

Risk factors of worsening headache	No affect at all	Mostly no affect	Neutral	Moderate affect	Strongly affect
Long online class hours	5 (15.6%)	11 (34.4%)	9 (28.1%)	6 (18.8%)	1 (3.1%)
Difficulty in online class	10 (31.3%)	5 (15.6%)	7 (21.9%)	9 (28.1%)	1 (3.1%)
Increased digital devices usage time	1 (3.1%)	2 (6.3%)	8 (25%)	11 (34.4%)	10 (31.3%)
Irregular or decreased sleep time	3 (9.4%)	5 (15.6%)	11 (34.4%)	9 (28.1%)	4 (12.5%)
Changed eating habits	6 (18.8%)	8 (25%)	7 (21.9%)	8 (25%)	3 (9.4%)
Decreased exercise time	3 (9.4%)	3 (9.4%)	7 (21.9%)	13 (40.6%)	6 (18.8%)
Reduced frequency of going out	5 (15.6%)	5 (15.6%)	4 (12.5%)	8 (25%)	10 (31.3%)
Decreased leisure activity	5 (15.6%)	3 (9.4%)	9 (28.1%)	8 (25%)	7 (21.9%)
Wearing a mask for a long time	1 (3.1%)	3 (9.4%)	5 (15.6%)	3 (9.4%)	20 (62.5%)
Stress from social distance rules	4 (12.5%)	6 (18.8%)	8 (25%)	8 (25%)	6 (18.8%)
Social isolation	5 (15.6%)	2 (6.3%)	7 (21.9%)	8 (25%)	10 (31.3%)
Anxiety about COVID-19 infection	7 (21.9%)	9 (28.1%)	6 (18.8%)	6 (18.8%)	4 (12.5%)
Depressed mood	9 (28.1%)	7 (21.9%)	6 (18.8%)	7 (21.9%)	3 (9.4%)
Conflicts with family	9 (28.1%)	5 (15.6%)	10 (31.3%)	2 (6.3%)	6 (18.8%)

COVID-19, coronavirus disease.

## Discussion

Headache in children and adolescents is related to genetic factors; psychological factors such as stress, anxiety, depression, behavioral disorders, and obsessive-compulsive disorder; lifestyle factors such as sleep or diet; and secondary factors such as infections or tumors (14, 15). Since the spread COVID-19, South Korea has confirmed 30.5 million cases of COVID-19 infection until February 2023, with a total of 33,977 deaths. Thus, our daily life styles have undergone significant changes after COVID-19 pandemic, affecting various fields such as politics, economy, society, education, and medicine. To prevent the spread of COVID-19, interpersonal contact has been restricted at the national level (16). This could have imposed a stressful situation, especially for children and adolescents who spend most of their days in groups, such as schools or private tutoring centers. Meade et al. reviewed recent studies examining mental health outcomes in the general population of children and adolescents during the COVID-19 pandemic (17). Evidence suggests that social isolation and sedentary behaviors contribute to mental health problems in children and adolescents. These can be confirmed to some extent by the overlap with the triggers and aggravating factors of headache observed in our study. A recent study also reported an increase in the frequency of migraine headache after the COVID-19 pandemic. In the results of the logistic regression analysis, younger age, mood deterioration, sleep problems score were significantly associated with worsening headache (18). Our study investigated the impact of daily life and lifestyle changes caused by the pandemic on pediatric patients with primary headache.

The COVID-19 infection may be considered as an aggravating factor in the course of primary headache. However, there is still a lack of data to support this hypothesis. A previous study reported the characteristics of COVID-19-related headache as a result of a web-based survey (19). They compared the participants with and without COVID-19 with respect to the presence or absence of previous episodes of headaches before the COVID-19 pandemic and headache characteristics. They reported that the presence of bilateral headache, duration ≥72 h, male sex, analgesic resistance, gastrointestinal symptoms, and anosmia/ageusia led to an increased risk of experiencing headache related to COVID-19 infection. However, they could not present data on the direct effect of COVID-19 on the exacerbation of primary headache. This may be attributed to the difficulty in properly collecting data during the lockdown period owing to the clinical characteristics of primary headache, which can be examined only through repeated observations over a long period of time. Therefore, we prepared questionnaire investigating the exacerbating factors of COVID-19 on the patients with primary headache.

One of the biggest changes in daily life since the pandemic is wearing masks. Due to the nature of the COVID-19, which is spread by droplets, students were taught to cover both their nose and mouth with a mask in the class. In our study, students wore masks for an average of 7.5 ± 2.2 h/day and 6.1 ± 1.0 days/week, which implies that they wore masks for about half of their waking hours. In a study by Toksoy et al. (20) targeting 375 healthcare workers wearing masks, 67.5% of the 114 people who had previously had headaches responded that their headaches had worsened since wearing masks; moreover, after the pandemic, new episodes of headache occurred in 30.9% of the surveyed people. Factors such as mechanical compression, hypoxemia, and hypercarbia triggered by personal protective equipment have been implicated in the pathogenesis of headache. Jonathan et al. (21) suggested that this equipment could trigger neural activity by stimulating the trigeminal and occipital nerve endings through compression and peripheral sensation. In another study, N95 masks were shown to alter the cerebral hemodynamics (22). Considering the role of the trigemino-vascular system activation in the pathophysiology of migraine (23–25), it can theoretically be suggested that the use of personal protective equipment may lead to migraine activation. In the case of children and adolescents, it may be difficult to find a mask that is suitable for their size, as the size of their face is smaller than that of the adults, and they are continuously growing. Therefore, if the mask does not fit well on the face, tighten the strap is tightened or a hook is used to attach the mask to the face so that the mask does not fall down. This can irritate the ears, jaw joints, and scalp and may affect the neural and vascular systems (21, 26, 27). Yuksel et al. compared the migraine worsening group to the stable or improved groups, and demonstrated the relationship between migraine exacerbation and mask type and number of masks (28). Recently, the Korean government has taken some measures to return to the pre-pandemic state, such as lifting of social distancing and the removal of the mandate on wearing of masks outdoors. However, wearing a mask indoors was maintained until January 2023. Moreover, in a situation where SARS-CoV-2 undergoes mutation and re-emergence, the mask wearing time of children and adolescents is not expected to show a significant change. Therefore, to reduce the occurrence and aggravation of headache caused by wearing masks, it is important to wear a mask that has a suitable size matching that of the face and to avoid tightening the straps or causing excessive stimulation to the face and head.

In our survey, the patients reported increased time spent using digital devices as another important factor that had an impact on headache aggravation. As the age of first encounter of digital devices, such as smartphones is younger, children and adolescents spend a considerable amount of time in front of the screen even before the pandemic (29). As various restrictions were placed on spending leisure time due to the pandemic, screen time increased. Moreover, with the school classes being conducted online, many hours of the day were spent with digital devices. Wehbe et al. (30) revealed that during current confinement, 95.6% of the adolescents spent 9 or more hours/day on screen during the weekdays, and most adolescents used some form of screen unitl the time they fall asleep. The majority (94%) of the studied adolescents reported having headache at least a few times a month. The triggers for headaches included excessive screen time (17.9%), lack of sleep (23.3%), and stress (22.1%). Cheung et al. (31) also stated that early adolescents who spent more time using display devices during COVID-19 had significantly poorer health-related quality of life outcomes. To reduce the aggravation of headache caused by the increased screen time, the efforts of the parents are needed to minimize the screen time as much as possible and refrain from spending leisure time using digital devices.

Operto et al. compared the mean scores of the Parenting Stress Index (PSI) and the Child Behaviour Check List (CBCL) before and after the pandemic in children and adolescents with neuropsychiatric disorder (32). They suggested that the COVID-19 pandemic led to increase in internalizing and externalizing symptoms, and a higher level of stress in parents can be related to the internalizing symptoms of their children (32). Primary headaches has a higher prevalence of internalizing symptoms such as anxiety, depression, and somatic complaints (33). However, our study confirmed that “depressive mood” and “conflict with family” were relatively less affected by COVID-19 than other items because most of the participants were elementary and middle school students. Our second questionnaire aimed at checking the Ped-MIDAS scores and headache patterns 3 months after the first survey; it was conducted while social distancing was still being implemented. The questionnaire results revealed that more patients had decreased pain scores, Ped-MIDAS scores, and headache frequencies. From a completely different perspective than previous studies, Dallavalle et al. suggested that children and adolescents experienced a significant reduction in migraine symptoms during lockdown compared to that in the period before the lockdown, because of the positive effect of reducing the environmental challenges and daily pressures (34).

The first limitation of our study is that it was a single-center study with a small sample size. In addition, selection bias in patient recruitment could not be completely excluded, where only patients with primary headache visited the outpatient clinic when hospitals access was deemed difficult due to social distancing during the COVID-19 pandemic. Moreover, the results could be affected by recall bias because we used questionnaires that relied on recalling past memories of the symptoms experienced by the patients and opinions about the underlying risk factors. The strength of our study is reflected in comprising detailed scores for each variable, which differs from retrospective chart reviews. It also provides detailed results using a Likert scale that can objectify subjective questions about the aggravating risk factors of primary headache during a pandemic. In addition, accurate diagnosis and high reliability of the answers can be expected because the questionnaire was completed during the treatment phase by the headache specialist.

## Conclusion

In conclusion, our study revealed that the COVID-19 pandemic situation can introduce various changes in the daily lives of children, which in turn can affect the management of headache. By examining the feedback regarding the thoughts of the patients on the impact of the current pandemic environment, patient counseling on the precautions and management can be conducted in advance in the case of repeated lockdown in the future.

## Data Availability

The original contributions presented in the study are included in the article/**Supplementary Material**, further inquiries can be directed to the corresponding author.
