# Supplementary material for: The impact of the COVID-19 pandemic era on children with primary headache: a questionnaire survey study and literature review
Source: Front Pediatr. 2023 Jul 10;11:1179979. doi: 10.3389/fped.2023.1179979 (PMC10364439; doi:10.3389/fped.2023.1179979)
Supplement: Supplementary file 1 [file Datasheet1.pdf]

1. Supplementary Data

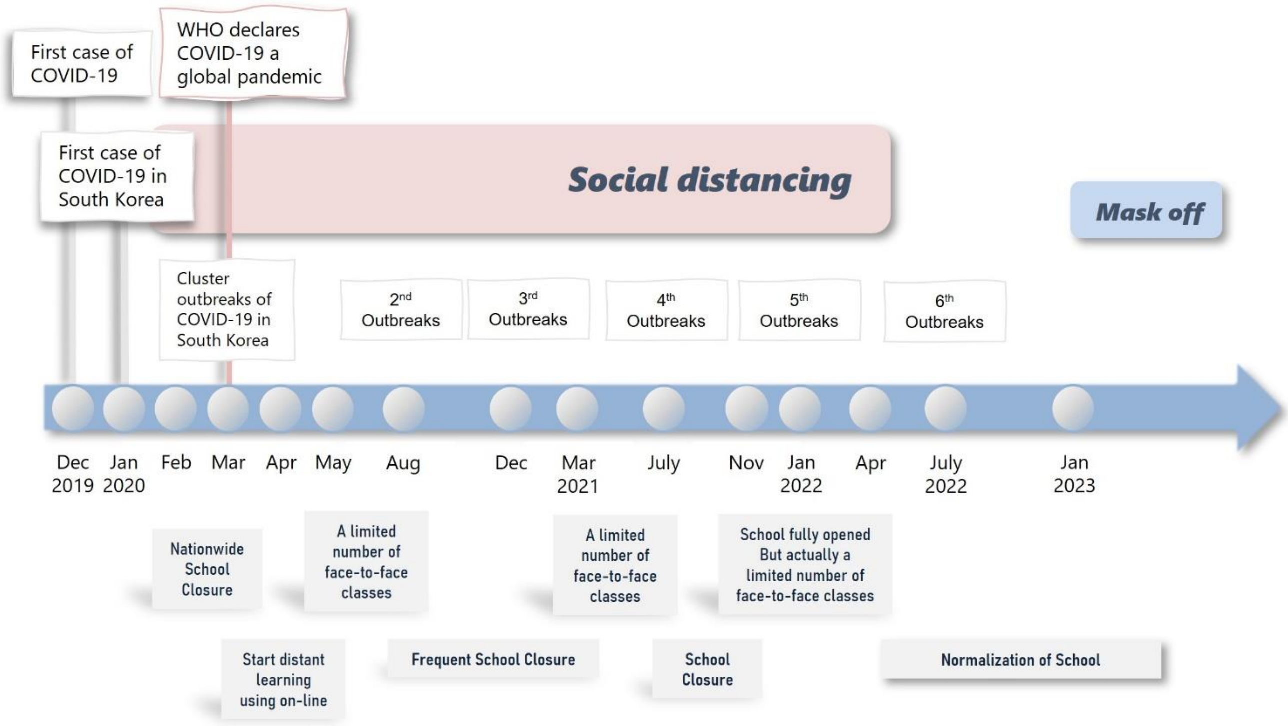

2. Supplementary Data

1st Questionnaire for children with primary headache in Korean

코로나 19 팬데믹 시대에 소아청소년의 두통 발현 양상에 관한 설문 조사

<1 차 설문지>

이 연구는 식사, 수면, 운동 시간의 변화, 온라인 강의 시청으로 인해 늘어난 컴퓨터 사용 증가, 교우관계 및 가족 관계의 변화로 인한 영향, 스마트폰, 게임 사용 시간 변화 등 코로나 19 팬데믹으로 변화된 전반적인 일상생활이 소아청소년의 두통 발현 양상에 미치는 영향에 대해 알아보고자 귀하의 생각을 물어보는 설문 조사입니다. 본 설문조사의 내용에는 환자 개인임을 식별할 수 있는 의료 정보는 전혀 포함되지 않고 설문조사 결과는 학문적 목적 외에는 사용되지 않습니다. 설문지 작성에 관한 동의서를 작성하신 분들에 한하여 각 문항별로 **해당하는 답변에 ☑ 표시해주십시오.**

작성일 : 년 월 일

답변자 : □ 보호자 (부/모) □ 환자 본인 □ 보호자와 환자 함께 작성

1. 환자의 출생 년도를 고르십시오.

□ 2013 □ 2012 □ 2011 □ 2010 □ 2009 □ 2008

□ 2007 □ 2006 □ 2005 □ 2004 □ 2003

2. 환자의 학년을 고르십시오.

□ 초등학교 3 학년 □ 초등학교 4 학년 □ 초등학교 5 학년 □ 초등학교 6 학년

□ 중학교 1 학년 □ 중학교 2 학년 □ 중학교 3 학년

□ 고등학교 1 학년 □ 고등학교 2 학년 □ 고등학교 3 학년 □ 기타 ( )

3. 환자의 성별을 고르십시오.

□ 남자 □ 여자

4. 2020 년 1 월 이전 반복적인 두통이 있었습니까?

□ 예 (5 번 질문부터 모두 답해주세요) □ 아니오 (7 번 질문부터 답해주세요)

5. 2020 년 1 월 이전 반복적인 두통이 있었다면 첫 두통이 있었던 것은 몇 년도였습니까?

□ 2019 년 □ 2018 년 □ 2017 년 □ 2016 년 □ 2015 년 □ 2014 년

□ 2013 년 □ 2014 년 □ 2013 년 □ 2012 년 □ 2011 년

6. 2020 년 1 월 이전 반복적인 두통이 있었다면 두통은 얼마나 자주 있었습니까?

□ 일 년에 1-5 번 □ 일 년에 6-12 번 □ 매 달 1-3 회 □ 매 달 4-7 회

□ 매 달 8-14 회 □ 매 달 15-30 회

7. 2020 년 1 월 이후 반복적인 두통이 있었습니까?

□ 예 (8 번 질문부터 모두 답해주세요)

□ 아니오 (이번이 첫 두통인 경우 11 번 질문부터 답해주세요)

8. 2020 년 1 월 이후 반복적인 두통이 있었다면 **첫 두통**이 있었던 때는 언제였습니까?

2020 년 □ 1 월 □ 2 월 □ 3 월 □ 4 월 □ 5 월 □ 6 월 □ 7 월 □ 8 월 □ 9 월 □ 10 월

□ 11 월 □ 12 월

2021 년 □ 1 월 □ 2 월 □ 3 월 □ 4 월 □ 5 월 □ 6 월 □ 7 월 □ 8 월 □ 9 월 □ 10 월

□ 11 월 □ 12 월

2022 년 □ 1 월 □ 2 월 □ 3 월 □ 4 월 □ 5 월 □ 6 월 □ 7 월 □ 8 월 □ 9 월 □ 10 월

□ 11 월 □ 12 월

9. 2020 년 1 월 이후 반복적인 두통이 있었다면 두통은 얼마나 자주 있습니까?

□ 일년에 1-5 번 □ 일년에 6-12 번 □ 매달 1-3 회 □ 매달 4-7 회

□ 매달 8-14 회 □ 매달 15-30 회

10. 두통 관련하여 최근 6 개월간 해당하는 항목이 있으면 모두 고르십시오.

□ 새로운 시작한 두통 □ 점점 심해지는 두통 □ 누우면 악화되는 두통

□ 자다가 깰 정도의 새벽 또는 아침 두통 □ 갑자기 천둥치는 듯한 극심한 두통

□ 두통 시 의식변화 또는 실신, 경련 등 증상 동반 □ 기침 시 두통

◆ 11 번 질문부터는 최근 평소 두통의 빈도, 강도, 양상에 대한 질문입니다. 모두 응답해 주십시오.

11. 두통이 시작되면 얼마나 지속됩니까?

□ 수 초 ~ 수 분 □ 수 십분 ~ 2 시간 미만 □ 2 시간 이상 ~ 12 시간 미만

□ 12 시간 이상 ~ 24 시간 미만 □ 24 시간 이상 ~ 72 시간 미만 □ 72 시간 이상

12. 두통 시 주로 아픈 머리 부위가 어디인가요?

□ 한쪽 □ 양쪽 □ 이마 □ 눈 주위 □ 관자놀이 □ 뒤통수 □ 목

13. 머리가 아픈 양상을 고르십시오.

□ 박동성 (맥박이 뛰는 듯이 욱씬욱씬, 지끈지끈, 쿡쿡 쑤시는 양상)

□ 띠로 머리를 조이는 듯함 (꽉 누름, 묵직함)

□ 심하게 화끈거리거나 찌르는듯한 통증

□ 기타 ( )

14. 일상적인 신체활동 (걷거나 계단 오르기 등)에 의해 두통이 악화되거나 이런 일상 행동을 두통 때문에 피하게 됩니까?

□ 예 □ 아니오

15. 두통 시작 후 가장 아플 때 어느 정도로 아픈가요?

(얼굴 그림 점수에 동그라미 쳐 주십시오.)

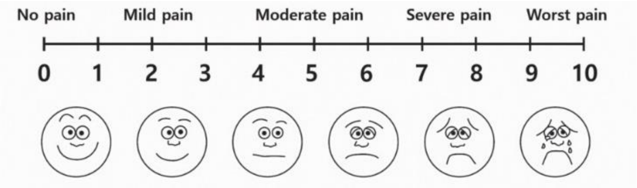

16. 두통 시 동반되는 증상을 모두 고르십시오.

□ 속이 울렁거린다 □ 토한다 □ 밝은 빛에 두통이 악화된다 □ 큰 소리에 두통이 악화된다

□ 특정 냄새가 싫다 □ 한쪽 눈의 충혈/눈물 □ 한쪽 코 막힘/ 콧물 □ 한쪽 눈의 부종

□ 한쪽 이마/얼굴의 땀

17. 두통 시작 전, 후 1 시간 사이에 뭔가 조짐(aura)증상이 있으면 모두 고르십시오.

□ 없다 □ 반짝반짝 빛이나 지그재그 선이 보인다 □ 눈 앞이 부분적으로 깜깜해진다 또는 까맣게 보인다 (암점) □ 감각 이상 (팔, 다리, 몸통) □ 일시적 말하기 오류(착어증), 언어 이해 어려움, 입이나 혀 주위 감각 이상으로 발음 곤란

18. 다음 중 본인의 두통을 유발이나 악화 시키는 것 같다고 생각하는 요인을 모두 표시하여 주십시오.

□ 학업 스트레스 □ 대인관계 스트레스 □ 수면 부족 □ 늦잠 □ 과식 □ 불규칙한 식사 □ 굶을 때 □ 냄새 □ 시끄러운 소리 □ 추위 □ 더위 □ 햇빛 □ 운동 □ 높은 습도 □ 건조한 날씨 □ 음식 (종류:) □ 커피 □ 술 □ 담배 □ 호르몬 변화 (생리 주기 등) □ 기타 ( )

19. 최근 3 개월간 두통 때문에 학교 수업 또는 학원 수업(대면수업, 온라인 수업 등)을 완전히 빠졌던 날은 몇 일이나 됩니까?

답변: 총 92 일 중 일

20. 최근 3 개월간 두통 때문에 학교 수업 또는 학원 수업 중 중간에 조퇴한 적은 몇 일이나 됩니까? (19 번 항목에 해당하는 날들은 제외)

답변: 총 92 일 중 일

21. 최근 3 개월간 두통 때문에 학교 수업 또는 학원 수업 중 학습 능률이 절반 이하로 감소한 날은 몇 일이나 됩니까? (19,20 번 항목에 해당하는 날들은 제외)

답변: 총 92 일 중 일

22. 최근 3 개월간 두통 때문에 집에서 일을 (숙제, 청소 등 간단한 집안일 등) 할 수 없었던 날은 몇 일이나 됩니까?

답변: 총 92 일 중 일

23. 최근 3 개월간 두통 때문에 다른 활동에 (놀이, 외출, 운동, 여행 등) 참여하지 못하였던 날은 몇 일이나 됩니까?

답변: 총 92 일 중 일

24. 최근 3 개월간 두통 때문에 다른 활동에 (놀이, 외출, 운동, 여행 등) 부분적으로 참여하여야 했던 날은 몇 일이나 됩니까? (24 번 항목에 해당하는 날들은 제외)

답변: 총 92 일 중 일

25. 두통 시 진통제 (타이레놀, 부르펜 등) 복용하면 두통 호전에 도움이 됩니까?

□ 예 □ 절반 정도는 효과가 있는 것 같다 □ 효과 없다

26. 최근 한달 간 평균적으로 진통제 복용을 얼마나 하였습니까?

□ 복용 안함 □ 한 달에 1-3 번 □ 한 달에 4-8 번 (일주일에 한두번)

□ 일주일에 세네 번 □ 한 달에 15 일 이상 또는 거의 매일

27. 두통 시 복용해본 진통제 종류를 모두 적어보세요. (타이레놀, 부르펜, 나프록센, 트립탄 등)

( )

28. 두통이 유발될 수 있는 다른 병을 진단 받거나 앓은 적이 있습니까?

□ 예 (진단명 :) □ 아니오

29. 태어나서 지금까지 입원한 적이 있거나, 다른 병을 진단 받거나 앓은 적이 있습니까?

□ 예 (진단명 :) □ 아니오

30. 가족 중 두통 환자가 있습니까?

□ 예 (관계: , 진단명 또는 양상 :) □ 아니오

◆ 31 번 질문부터는 코로나 19 감염과 두통과의 관련성에 대한 생각에 대한 내용입니다. 해당하실 경우 표시해 주십시오.

31. 코로나 19 팬데믹 이후 지금까지 코로나 19 접촉으로 인한 자가격리를 시행한 적이 있습니까?

□ 예 (날짜 년 월 일 ~ 년 월 일) □ 아니오

32. 코로나 19 팬데믹 이후 지금까지 코로나 19 를 진단 받은 적이 있습니까?

□ 예 (날짜 년 월 일) □ 아니오

33. 코로나 19 에 걸린 적이 있었다면 그 기간 동안 동반된 증상 및 합병증이 있었습니까? 해당되는 것을 <u>모두 고르시오.</u>

□ 무증상

□ 열 □ 콧물 □ 몸살 기운 □ 기침 □ 호흡곤란 □ 폐렴 □ 흉통 □ 심근염 □ 부정맥, 심계항진 □ 후각소실 □ 맛 소실 (미각 소실) □ 구토 □ 설사 □ 식욕 감퇴 □ 피부 발진 □ 점상출혈 (혈소판 감소) □ 혈전증 □ 두통 □ 어지럼증 □ 수면 장애 □ 우울증 □ 감각 이상 □ 경련 □ 뇌수막염 □ 뇌염 □ 뇌경색 □ 패혈증 □ 근력 약화, 보행장애 □ 탈모 □ 횡문근융해증 □ 신장기능이상 (단백뇨, 혈뇨, 급성 신부전 등)

34. 코로나 19 에 걸린 적이 있었다면 전 후로 원래 있던 반복적인 두통의 증상이 변화하였습니까?

□ 예 (원래 증상: → 변화 후 증상:) □ 아니오

◆ 35 번 질문부터는 코로나 19 팬데믹 전후의 일상생활과 두통과의 관련성에 대한 생각에 대한 내용입니다. **<u>모두 응답해 주십시오.</u>**

35. 코로나 19 팬데믹 이후 일상생활의 변화에 대해 각 항목에 해당하는 내용을 <u>모두 고르십시오.</u>

- 운동 시간 변화 (□ 증가 □ 비슷 □ 감소)
- 수면 시간 변화 (□ 규칙적 □ 불규칙적 □ 증가 □ 감소 □ 비슷)
- 식사 변화 (□ 규칙적 □ 불규칙적 □ 식사량 증가 □ 식사량 감소 □ 배달음식 증가 □ 배달음식 감소)
- 체중 변화 (□ 증가 □ 비슷 □ 감소)

- 마스크 착용 시간 : 일주일에____일 , 하루 평균 ___시간 정도

- 코로나 19 이후 수업 시간 온라인 학교 수업 하루 평균 _____________시간

온라인 학원 수업 하루 평균 _____________시간 (일주일에 일)

- 코로나 19 이전 수업 시간 학교 수업 하루 평균 _____________시간

학원 수업 하루 평균 _____________시간 (일주일에 일)

- 온라인 수업시간 증가로 인한 학습의 어려움 (□있다 □ 없다_잘 적응하였다)

- 스마트폰 사용 시간 코로나 19 전 하루 평균 _________시간

코로나 19 후 하루 평균 _________시간

- 컴퓨터, PC, 게임기, TV 등 사용 시간 (학습 제외)

코로나 19 전 하루 평균 _________시간

코로나 19 후 하루 평균 _________시간

- 외출 빈도 (학교, 학원 외) 코로나 19 전 일주일에 ___일

코로나 19 후 일주일에 ___일

- 여가생활 빈도 (가족여행, 문화생활 등) 코로나 19 전 한 달에 ___일

코로나 19 후 한 달에 ___일

- 가정 경제 사정 (□ 코로나 19 전과 비슷하게 유지 □ 코로나 19 이후 어려워짐)

- 가정에서 수업이나 학습 외에 시간에 주로 무엇을 하며 지내나요?

( )

36. **<u>코로나 19 팬데믹이</u>** <u>두통을 악화시킬 수 있는 요인</u>에 <u>미치는 영향</u>과 관련하여 본인이 생각하는 정도에 따라 해당 점수 위에 동그라미 체크를 해주십시오.

1=전혀 상관없다 2=대체로 상관없다 3= 보통이다 4= 약간 영향을 받았다 5=매우 크게 영향을 받았다

긴 온라인 교육 시간으로 인한 스트레스

1------------2------------3------------4------------5

온라인 교육 수업으로 인한 학습의 어려움 스트레스

1------------2------------3------------4------------5

스마트폰, 컴퓨터, 노트북, TV 사용시간의 증가

1------------2------------3------------4------------5

불규칙한 수면 또는 수면 부족

1------------2------------3------------4------------5

불규칙한 식사 또는 과식

1------------2------------3------------4------------5

운동 부족

1------------2------------3------------4------------5

외출 횟수 감소

1------------2------------3------------4------------5

여가 및 문화 생활 감소

1------------2------------3------------4------------5

장시간의 마스크 착용

1------------2------------3------------4------------5

거리두기 등의 국가적 상황에 대한 스트레스

1------------2------------3------------4------------5

친구들과 만나지 못하여 생기는 사회적 고립 환경

1------------2------------3------------4------------5

코로나 19 감염에 대한 두려움, 불안감

1------------2------------3------------4------------5

우울감

1------------2------------3------------4------------5

장기간 가정생활로 인한 잦은 가족 마찰, 가족 스트레스

1------------2------------3------------4------------5

3. Supplementary Data

2nd Questionnaire for children with primary headache in Korean

코로나 19 팬데믹 시대에 소아청소년의 두통 발현 양상에 관한 설문 조사

< 2 차 설문지 >

이 2 차 설문지는 이전 본인/보호자께서 작성하여 제출해 주셨던 1 차 설문지에 이어지는 연구 내용으로 지난 3 개월간 코로나 19 팬데믹으로 인한 전반적인 일상생활 변화 상태와 두통 발현 양상에 대해 알아보고자 귀하의 생각을 물어보는 설문 조사입니다. 본 설문조사의 내용에는 환자 개인임을 식별할 수 있는 의료정보는 전혀 포함되지 않고 설문조사 결과는 학문적 목적 외에는 사용되지 않습니다. 설문지 작성에 관한 동의서를 작성하신 분들에 한하여 각 문항별로 **해당하는 답변에 ☑ 표시해주십시오.** 작성하신 후 **소아청소년과 외래에 전달** 주시면 감사하겠습니다. 서면 제출하지 못하시는 상황 발생시 1 차 설문지에서 작성해 주셨던 전화 번호로 연락 드려 구두 설문 조사 형태로 진행될 예정입니다.

작성 예정일 : 년 월 일

실제 작성일 : 년 월 일

답변자 : □ 보호자 □ 환자 본인 □ 보호자와 환자 함께 작성

1. 지난 3 개월 간 있었던 평균 두통 횟수를 고르십시오.

□ 없었음 □ 한 달에 한 두번 미만 □ 일주일에 한 두번 □ 일주일에 두 세번

□ 한 달에 15 일 이상 또는 매일

2. 지난 3 개월 간 두통 시 평균적인 두통 점수는 몇 점이었습니까? (점)

(얼굴 그림 점수에 동그라미 쳐 주십시오.)

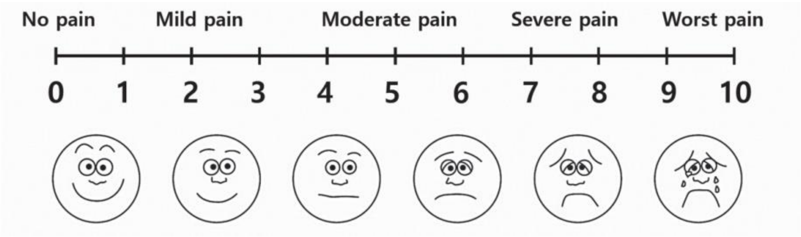

3. 최근 3 개월간 두통 때문에 학교 수업 또는 학원 수업(대면수업, 온라인 수업 등) 을 완전히 빠졌던 날은 몇 일이나 됩니까?

답변: 총 92 일 중 일

4. 최근 3 개월간 두통 때문에 학교 수업 또는 학원 수업 중 중간에 조퇴한 적은 몇 일이나 됩니까? (19 번 항목에 해당하는 날들은 제외)

답변: 총 92 일 중 일

5. 최근 3 개월간 두통 때문에 학교 수업 또는 학원 수업 중 학습 능률이 절반 이하로 감소한 날은 몇 일이나 됩니까? (19,20 번 항목에 해당하는 날들은 제외)

답변: 총 92 일 중 일

6. 최근 3 개월간 두통 때문에 집에서 일을 (숙제, 청소 등 간단한 집안일 등) 할 수 없었던 날은 몇 일이나 됩니까?

답변: 총 92 일 중 일

7. 최근 3 개월간 두통 때문에 다른 활동에 (놀이, 외출, 운동, 여행 등) 참여하지 못하였던 날은 몇 일이나 됩니까?

답변: 총 92 일 중 일

8. 최근 3 개월간 두통 때문에 다른 활동에 (놀이, 외출, 운동, 여행 등) 부분적으로 참여하여야 했던 날은 몇 일이나 됩니까? (24 번 항목에 해당하는 날들은 제외)

답변: 총 92 일 중 일

9. 두통 시 진통제 (타이레놀, 부르펜 등) 복용하면 두통 호전에 도움이 됩니까?

□ 예 □ 절반 정도는 효과가 있는 것 같다 □ 효과 없다

10. 최근 한달 간 평균적으로 진통제 복용을 얼마나 하였습니까?

□ 복용 안함 □ 한 달에 1-3 번 □ 한 달에 4-8 번 (일주일에 한두번)

□ 일주일에 세네 번 □ 한 달에 15 일 이상 또는 거의 매일

11. 두통 시 복용해본 진통제 종류를 모두 적어보세요. (타이레놀, 부르펜, 나프록센, 트립탄 등)

( )

12. 지난 3 개월 동안 코로나 19 접촉으로 인한 자가격리를 시행한 적이 있습니까?

□ 예 (날짜 년 월 일 ~ 년 월 일) □ 아니오

13. 지난 3 개월 동안 코로나 19 를 진단 받은 적이 있습니까?

□ 예 (날짜 년 월 일) □ 아니오

14. 지난 3 개월 동안 코로나 19 에 걸린 적이 있었다면 그 기간 동안 동반된 증상 및 합병증이 있었습니까? 해당되는 것을 모두 고르시오.

□ 무증상

□ 열 □ 콧물 □ 몸살 기운 □ 기침 □ 호흡곤란 □ 폐렴 □ 흉통 □ 심근염 □ 부정맥, 심계항진 □ 후각소실 □ 맛 소실 (미각 소실) □ 구토 □ 설사 □ 식욕 감퇴 □ 피부 발진 □ 점상출혈 (혈소판 감소) □ 혈전증 □ 두통 □ 어지럼증 □ 수면 장애 □ 우울증 □ 감각 이상 □ 경련 □ 뇌수막염 □ 뇌염 □ 뇌경색 □ 패혈증 □ 근력 약화, 보행장애 □ 탈모 □ 횡문근융해증 □ 신장기능이상 (단백뇨, 혈뇨, 급성 신부전 등)

15. 지난 3 개월 동안 코로나 19 에 걸린 적이 있었다면 전 후로 원래 있던 반복적인 두통의 증상이 변화하였습니까?

□ 예 (원래 증상: → 변화 후 증상:) □ 아니오

16. **코로나 19 팬데믹이** 두통을 악화시킬 수 있는 요인에 미치는 영향과 관련하여 본인이 생각하는 정도에 따라 해당 점수 위에 동그라미 체크를 해주십시오.

1=전혀 상관없다 2=대체로 상관없다 3= 보통이다 4= 약간 영향을 받았다 5=매우 크게 영향을 받았다

긴 온라인 교육 시간으로 인한 스트레스

1------------2------------3------------4------------5

온라인 교육 수업으로 인한 학습의 어려움 스트레스

1------------2------------3------------4------------5

스마트폰, 컴퓨터, 노트북, TV 사용시간의 증가

1------------2------------3------------4------------5

불규칙한 수면 또는 수면 부족

1------------2------------3------------4------------5

불규칙한 식사 또는 과식

1------------2------------3------------4------------5

운동 부족

1------------2------------3------------4------------5

외출 횟수 감소

1------------2------------3------------4------------5

여가 및 문화 생활 감소

1------------2------------3------------4------------5

장시간의 마스크 착용

1------------2------------3------------4------------5

거리두기 등의 국가적 상황에 대한 스트레스

1------------2------------3------------4------------5

친구들과 만나지 못하여 생기는 사회적 고립 환경

1------------2------------3------------4------------5

코로나 19 감염에 대한 두려움, 불안감

1------------2------------3------------4------------5

경제적 어려움

1------------2------------3------------4------------5

우울감

1------------2------------3------------4------------5

장기간 가정생활로 인한 잦은 가족 마찰, 가족 스트레스

1------------2------------3------------4------------5
